# Assessing Minority Stress and Physiological Response Through Ecological Momentary Assessment and Sensors: Protocol for a Feasibility and Acceptability of the Stress and Heart Pilot Study

**DOI:** 10.2196/68733

**Published:** 2025-10-15

**Authors:** Dulce Urueta Tapia, Heather L Corliss, Kang Hyuk Lee, Jerel P Calzo, Hee-Jin Jun

**Affiliations:** 1 San Diego State University Research Foundation San Diego, CA United States; 2 School of Public Health San Diego State University San Diego, CA United States; 3 San Diego State University Institute for Behavioral and Community Health San Diego, CA United States

**Keywords:** ecological momentary assessment, sensor, heart rate variability, stress, minority stress, sexual and gender minorities, acceptability, feasibility

## Abstract

**Background:**

LGBTQ+ (lesbian, gay, bisexual, transgender, queer, and other diverse sexual and gender identities) young adults may experience discrimination based on their sexual and gender minority status, which results in LGBTQ+ population health disparities. Although the health effects of minority stress have been studied for more than 30 years, most research relies on retrospective and cross-sectional designs. These methods limit the ability to establish causal links and to capture the fluctuating and time-sensitive nature of stress responses. In contrast, ecological momentary assessment (EMA) and wearable sensors allow for real-time tracking of stress exposures and their resulting physiological effects, enhancing data accuracy and ecological validity.

**Objective:**

The EMA and sensor protocol of the Stress and Heart Study was developed to capture real-time physiological responses to minority stress experiences among LGBTQ+ young adults. This pilot study aims to evaluate the acceptability and feasibility of the Stress and Heart Study protocol.

**Methods:**

Participants who identified as LGBTQ+ between the ages of 18 and 30 years who reported experiencing LGBTQ+ discrimination in the past 30 days in a screening survey were invited to participate in a 2-week EMA and sensor study. Participants received 4 daily EMA surveys and one end-of-day (EOD) survey on their smartphones, assessing general and minority stress experiences, positive and negative emotional states, and substance use. Heart rate variability was continuously recorded using a wearable sensor. Upon completion, participants completed a short exit survey to evaluate their study experience and satisfaction.

**Results:**

Twenty participants aged 18 to 27 (mean 21.7, SD 2.6) years were enrolled, representing diverse sexual orientations (8 lesbian/gay, 2 bisexual, 4 pansexual, and 6 queer), gender identities (10 cisgender, 3 transgender, and 7 non-binary), and racial/ethnic backgrounds (9 non-Hispanic [NH] White, 5 Latinx, 2 NH Black, and 4 NH Asian). Participants completed 89.4% (1001/1120) of EMA daily surveys and 92.1% (258/280) of EOD surveys. On average, participants wore the sensor for 74.6% (SD 24%) of the expected time (179/240 hours). The overall EMA daily and EOD surveys completion rate was 89.9% (1259/1400), with individual participation ranging from 70% (49/70) to 98.6% (69/70). A total of 85% (n=17) of participants reported wearing the sensor daily. In the exit survey, all participants indicated that the study’s time commitment met their expectations. Additionally, 90% (n=18) of participants reported that the sensor was comfortable and that the EMA app was user-friendly, with appropriately timed questions.

**Conclusions:**

This study supports the feasibility and acceptability of the Stress and Heart Study protocol using smartphones and wearable sensors to collect real-time data on minority stress experiences and physiological response. Further research is needed to validate the use of this protocol in larger observational and intervention studies aimed at addressing the adverse health impacts of minority stress among LGBTQ+ populations.

## Introduction

Cardiovascular disease (CVD), which includes conditions such as coronary heart disease, cerebrovascular disease, and deep vein thrombosis that can lead to heart attack or stroke, is the leading cause of morbidity and mortality in the United States [1]. Evidence suggests that certain populations, including racial and ethnic minorities, individuals with low socioeconomic status, and sexual and gender minority (SGM) populations (ie, those who identify as lesbian, gay, bisexual, transgender, queer, and other diverse sexual and gender identities [LGBTQ+]), may be at increased risk for CVD [2-7]. However, research comparing the occurrence of CVD between LGBTQ+ and heterosexual cisgender populations has yielded mixed findings. Some studies report higher rates of CVD among specific LGBTQ+ subgroups [8-13] while others find similar or even lower rates [9-11,13,14].

Despite these mixed findings regarding CVD outcomes, a growing body of evidence indicates that LGBTQ+ populations experience poorer cardiovascular health (CVH) across multiple domains. These disparities include both clinical and physiological indicators (eg, hypertension, diabetes, high cholesterol, obesity, and depression) and behavioral risk factors (eg, tobacco and alcohol use, low physical activity, and poor sleep), compared with their heterosexual and cisgender counterparts [4,9,13,15-17].

A primary cause attributed to LGBTQ+ disparities in CVH and CVD is minority stress. Minority stress refers to the chronic stressors that LGBTQ+ individuals often encounter due to their stigmatized sexual orientation or gender identity [18-20]. The minority stress model posits that distal stressors (eg, discrimination, rejection, victimization, non-affirmation of identity) impact proximal stress processes (identity concealment, expectations of rejection/mistreatment, internalized stigma), leading to negative psychological, behavioral, and physiological outcomes. Research suggests that minority stress experiences are related to poorer CVH and greater risk for CVD [4,9,21,22].

Only a few studies have linked minority stress to CVD [9,23,24]. For example, one study found that greater exposure to discrimination and violence was associated with increased self-reported CVD among gay and bisexual adult men compared with heterosexual and cisgender counterparts [9]. Another study identified an indirect association between minority stress and CVD risk factors via reduced sense of mastery [24]. Additionally, transgender individuals have been shown to experience higher rates of cardiovascular morbidity and mortality, influenced by self-reported minority stress and gender-affirming hormone therapy [23]. These findings underscore the need to investigate physiological mechanisms linking minority stress to CVD outcomes.

Stress negatively affects CVH by disrupting the autonomic nervous system, which comprises the sympathetic and parasympathetic branches [25]. The sympathetic system triggers the “fight or flight” response, releasing cortisol and adrenaline to elevate heart rate and blood pressure, while the parasympathetic system promotes relaxation and recovery [25]. A balanced interaction between these systems is essential for physiological homeostasis. Chronic stress can disrupt this balance, leading to autonomic dysfunction and increasing the risk of CVD [26,27].

Heart rate variability (HRV), a validated noninvasive marker of autonomic nervous system function, reflects the variation in time intervals between heartbeats [28-30]. Low HRV reflects diminished parasympathetic activity and heightened sympathetic arousal, signaling autonomic imbalance and impaired homeostasis [25]. Chronic stress is associated with lower HRV, which in turn has been linked to increased CVD risk [31-35]. Low HRV also indicates reduced capacity to respond to both internal (eg, inflammation and chronic pain) and external (eg, discrimination, loneliness, and poverty) stressors, further contributing to physiological strain [29].

Given that LGBTQ+ individuals experience greater chronic stress compared with heterosexual and cisgender individuals, they may be more susceptible to lower HRV. However, studies explicitly connecting minority stress and HRV in LGBTQ+ populations are rare. A literature review identified only a few studies that examined this relationship [36,37]. Huebner [36] conducted a lab-based study involving 134 LGB (lesbian, gay, and bisexual) adults (mean age of 25.8 years) and found that exposure to an anti-gay social stressor during a laboratory task resulted in heightened cardiovascular reactivity—increased heart rate and cortisol—and reduced HRV, suggesting that minority stress can trigger immediate physiological changes that may contribute to long-term CVD risk.

Rosati et al [37] conducted another lab-based study comparing 19 LGB and 20 heterosexual White adults’ (mean age of 35 years) physiological responses to an emotional stress task. The LGB participants showed higher HRV compared with the heterosexual participants, contradicting the hypothesis that LGB people are more susceptible to low HRV. However, the same study also found that the LGB participants had higher total peripheral resistance (TPR)—an indicator of blood vessel constriction and increased strain on the cardiovascular system. This pattern is referred to as the “cardiovascular conundrum” [38,39], a physiological state characterized by both high TPR [38] and high HRV [39], which may result from chronic exposure to minority stress. Typically, high TPR is associated with low HRV. Given the limited and conflicting findings, additional research is needed to clarify how minority stress affects HRV and its implications for CVH.

In addition to laboratory-based research on the effects of stress on HRV, much of the literature to date has relied on cross-sectional designs [40-42] or longitudinal cohort studies [43]. While valuable, such studies often have recall bias and may fail to capture dynamic, real-world autonomic responses. Ecological momentary assessment (EMA) addresses these issues by collecting real-time data in participants’ natural environments, greatly reducing recall bias and improving ecological validity [44]. When combined with wearable sensors (eg, smartwatches), EMA enables noninvasive, high-frequency HRV monitoring [45-48]. Although the number of studies integrating EMA with wearable HRV is still relatively small, this area is rapidly expanding. Recent research has examined the association between momentary stress and HRV in daily life and generally found that increases in perceived stress are associated with lower HRV, although the magnitude of this relationship is often small. For example, Akbar et al [45], using a wrist-worn smartwatch, observed that HRV-derived stress peaks corresponded with self-reported stress during work and after-hours EHR tasks, and Martinez et al [47] reported that HRV features were significantly but weakly associated with perceived stress. Kim et al [46], using a chest-strap sensor, demonstrated a bidirectional pattern, where lower HRV predicted higher perceived stress, and stress predicted HRV decreases within 10 minutes.

These studies highlight that perceived stress is linked to short-term autonomic changes; yet, existing EMA + HRV research has largely focused on general daily stress and has not captured the excess minority stress uniquely experienced by LGBTQ+ populations. The Stress and Heart Study was designed to fill this gap by measuring both general daily stress and minority stress exposure in real time, alongside autonomic responses captured via noninvasive, wearable sensors. In this pilot study, we evaluate the feasibility and acceptability of this protocol in 20 LGBTQ+ young adults recruited from community settings, providing a foundation for future research on minority stress and cardiovascular health in real-world environments.

## Methods

### Project Overview

To achieve the study aims, we developed a methodological protocol to evaluate real-time assessments of general stress and minority stress experiences among SGM young adults based on minority stress theory [18-20]. We created an EMA questionnaire to measure stress experiences, mood, and substance use. Four focus group discussions were conducted with the target population to gather feedback to evaluate the face validity of the questionnaires and inform refinement of the questionnaires and the study procedures. We then recruited participants who agreed to participate in a 14-day EMA and sensor study (EMA/sensor study) to assess feasibility and acceptability. Participants self-reported momentary minority stress experiences, mood, and substance use using a smartphone-based app. Their HRV was measured using a wearable sensor.

### Target Population and Eligibility

Eligible participants met the following inclusion criteria: (1) aged 18-30 years; (2) identify as SGM; (3) reporting minority stress experiences at least “sometimes” on 2 or more occasions in the past year due to their SGM status; (4) agree to participate in focus group discussion for 2-hours (for focus group participants); (5) have a smartphone (for EMA/sensor study participants); and (6) agree to visit the study site twice and participate in the 2-week EMA/sensor study (for EMA/sensor study participants).

### Sample Size and Recruitment Procedures

We recruited 14 participants for the focus group and another 20 for the EMA/sensor study. There was no overlap in participants in the 2 samples. The focus groups were conducted via an internal review board (IRB)–approved Zoom (Zoom Video Communications) session and lasted 1 hour. Fourteen participants were sufficient to assess our measurement tools and gather feedback to refine our procedures. The inclusion of 20 participants in the EMA/sensor survey offered initial insights into the feasibility of recruitment and retention strategies for future scalable projects. The sample size was based on prior research with similar methods and practical budget constraints, including device costs and participant incentives [49]. With this sample size and study duration, we anticipated an 80% compliance rate, defined as completing 56 of 70 surveys over 14 days (about 2 weeks).

We used web-based and in-person methods to recruit participants, sharing our flyer with LGBTQ+ organizations in the San Diego area via email and in-person visits. The team also posted flyers on university bulletin boards and coffee shops frequented by students. The flyer outlined eligibility, compensation, and a US $50 random drawing for prescreening survey applicants via QR code or URL. After completing the prescreening, participants received a confirmation email regarding their availability for either the focus group Zoom session or the EMA/sensor study’s initial training at a private, nonclinical research facility affiliated with a university located in San Diego. To facilitate recruitment, individuals interested in the focus group or the EMA/sensor study who provided contact information were entered into a drawing for a US $50 gift card. A separate drawing was held for the Focus group and the EMA/sensor study. One winner was selected for each drawing and was notified via email.

### Selection of Baseline and EMA Measures

The study team met weekly from October 2020 to December 2022 to curate our assessment measures for both baseline and EMA data collection. Our weekly meetings focused on measure selections and the suitability of each measure for the baseline survey, the EMA questionnaire, or other aspects of data collection. Through a comprehensive literature review of articles indexed in MEDLINE (PubMed) and Google Scholar, we identified measures of SGM stress informed by the minority stress framework [18-20], as well as measures of mental health and substance use behavior. The search strategy included terminology related to methods (eg, EMA, sensor, smartwatch, and wristband), target population (eg, LGBTQ+, sexual orientation, gender identity, sexual and gender minority), substance involvement (eg, smoking, alcohol, marijuana, and drug consumption), and mental health (eg, depression, anxiety and affective states) relevant to our study objectives. Additional studies were also identified by examining the references cited in the literature. Emphasis was placed on identifying EMA studies or studies that addressed or included our target population.

To create concise EMA items, we reviewed validated measures to assess their validity and reliability in our target population. Factor analysis studies were examined to identify items with strong correlations to the construct, and the highest factor loading items were included in our Baseline and EMA protocol. For example, to assess sexual orientation–related stress, we selected items from the sexual orientation microaggression inventory-short form [50], retaining those with the highest factor loadings, such as “Heterosexism: overreact when talking about negative experience” (0.708) and “Societal Disapproval: don’t mind LGBQ people, they just shouldn’t be so public” (0.722). Once selected, the wording was adjusted to reflect the real-time, momentary nature of the measures (eg, since your last prompt). Our final measures are presented in Table 1. Focus group discussions were held to evaluate the face validity and acceptability of the selected items through cognitive assessment, a method that explores how participants interpret and respond to items [51].

**Table 1:** Baseline, ecological momentary assessment (EMA), and end-of-day (EOD) survey measures used in the Stress and Heart Study of LGBTQ+ (lesbian, gay, bisexual, transgender, queer, and other diverse sexual and gender identities) young adults, 2021-2022.

Variable				Baseline		EMA (4/day)					EOD
						Before Modification		After modification			
**Demographic characteristics**											
	Age			✓							
	Race/ethnicity			✓							
	Sexual orientation			✓							
	Gender identity			✓							
	Education			✓							
	Marital status			✓							
	Employment			✓							
	Income			✓							
**Mood and mental health**											
	Affective emotional states (I-PANAS-SF) [52]			✓		✓		✓			
	Generalized Anxiety Disorder-7 [53]			✓							
	PHQ-9 (Patient Depression Questionnaire) [54]			✓							
**Stress**											
	**Minority stress**										
		Everyday Discrimination Scale [55]	✓		✓		✓				
		Daily Heterosexist Experience Questionnaire (DHEQ) [56]	✓								
		Sexual Minority Adolescent Stress Inventory (SMASI) [57]	✓								
		Nebraska Outness Scale [58]	✓								
		Sexual Orientation Microaggression Inventory (SOMI-SF) [50]					✓				
	**General stress**										
		Ecological Momentary Assessment of Stressful Events [59]					✓				
		Current Stress Level [60]					✓				
		Perceived Stress Scale-4 [61]							✓		
**Substance use**											
	Alcohol use [62,63]			✓		✓		✓			
	Cigarette smoking, e-cigarette [62,63]			✓		✓		✓			
	Marijuana [62-64]			✓		✓		✓			
	Prescription drugs [63]			✓							
	Other drugs [63]									✓	

### Sensor Selection

For the sensor selection, we conducted a thorough search involving several approaches: reviewing previous studies to measure HRV, investigating commercially available sensors, analyzing comparison studies of sensors used for HRV measurement, and assessing the validity and reliability of HRV measurements [65,66]. Additionally, we considered ease of use, affordability based on the study budget, and compatibility with the EMA app. We evaluated the feasibility and desirability of different sensor types, including chest straps, wristbands, and pulse oximeters. Chest straps are known to be the most accurate way to measure HR, but they are often uncomfortable and burdensome for participants due to the need for chest electrodes [65,67]. Pulse oximeters are not currently designed for use by researchers overseeing multiple participants. On the other hand, wristbands can collect HR data continuously from the wrist. Given these considerations, we selected the Garmin Vivosmart 4 wristband [68,69]. This device offers several advantages, including accessing real-time data that matches EMA time frames. The Garmin software development kit provides real-time data via Bluetooth directly from the device to the EMA app on the phone and to ilumivu’s servers [70]. The Vivosmart 4 is lightweight, easy to wear, and has a long battery life, making it suitable for ambulatory assessment of HRV. It provides real-time heart rate and interbeat interval data, which are easily transmitted and integrated with the incoming EMA data.

### Focus Groups

The research team conducted 4 web-based focus group discussions between September 13 and October 15, 2021, to assess the validity of the candidate EMA measures and to enhance the survey’s acceptability and feasibility by gathering participant feedback. The research team and participants reviewed and discussed each survey question, leading to improvements. Participants also provided feedback on the Garmin Vivosmart4 watch, including their thoughts on adherence and barriers to wearing it while completing 5 daily EMA surveys on their smartphones over 2 weeks.

The focus group meetings were conducted via Zoom, a HIPAA (Health Insurance Portability and Accountability Act)-compliant video conferencing platform. Participants received confirmation emails with the meeting details, including the date, time, Zoom link, and focus group information. An Informed Consent Form documented the participants’ understanding of the study’s purpose and procedures, and their consent to participate. Participants reviewed and signed the digital consent form via Qualtrics before the session began. During the meetings, a research assistant presented the purpose of the study and the scheduling of the EMA and end-of-day (EOD) survey questions. Participants received a US $30 online gift card, chosen according to their preference, as an incentive for their participation. Feedback from the focus group discussions led to several important items being revised or added. Details of the modification are summarized in Table 2.

**Table 2:** Modifications to ecological momentary assessment (EMA) and end-of-day survey items based on focus group feedback in the stress and heart study of LGBTQ+ (lesbian, gay, bisexual, transgender, queer, and other diverse sexual and gender identities) young adults, 2021-2022.

Survey questions and original item				New or revised item
**Everyday Discrimination Scale [55]**				
	**Characteristics attributable to discrimination**			
		N/A^a^	Add gender identity	
		N/A	Add religion	
		N/A	Add age	
	**Place of discrimination**			
		N/A	Add LGBTQ community space	
**Substance use**				
	**Reasons and motivations**			
		Boredom	Release/manage boredom	
		Peer pressure	Socialize/confirm socially	
		N/A	Add influenced by alcohol/other substance	
**End-of-day survey**				
	**Daily device reminder**			
		N/A	Add at the end: “Please do not forget to charge your phone and watch and put the watch on in the morning.”	

^a^N/A: not applicable.

### EMA/Sensor Study

#### Study Procedure

Eligible participants who agreed to take part in the 2-week EMA/sensor study—conducted between April 23 and November 29, 2022—were invited to the study office located at SDSURF. The study was conducted in a private office room within the research facility to ensure a quiet, comfortable, and confidential environment for participants. Upon the participant’s arrival, a research assistant welcomed the participant and introduced the research team members. The assistant outlined the agenda for the first meeting and described the participant’s involvement over the next 2 weeks. The participant was then guided to review thoroughly the Informed Consent Form before signing it. Once the participant reviewed and signed the Informed Consent Form, the meeting continued with the participant filling out the baseline survey.

#### Baseline Survey

The 15-minute baseline Qualtrics survey assessed demographic characteristics and validated measures of mood and mental health, minority stress, general stress, and substance use. A complete list of baseline measures is presented in Table 1.

#### Training

Following completion of the baseline survey, the research assistant reviewed the study timeline. Participants were informed that, over the 2-week period, they would complete 4 EMA surveys and one EOD survey daily. The brief EOD survey captured experiences related to stress and substance use not covered in the EMA prompts (Table 1 displays survey content). To improve response rates and data reliability, we used an interval-contingent schedule for EMA prompts, delivering surveys at consistent, predetermined times throughout the day [44,71].

The research assistant worked with each participant to customize the 2-week EMA schedule, which consisted of 10 weekdays and 4 weekend days (2 weekend days each week). The survey typically started about one hour after waking and continued until bedtime, with approximately 3-hour intervals between surveys. The first prompt began within an hour of waking, and the remaining 3 followed at roughly 3-hour intervals. Start times could vary between weekdays and weekends to accommodate participants’ routines. For those with fixed commitments (eg, classes or work meetings), survey intervals were flexibly adjusted within a range of 2 to 4.5 hours. Across all participants, the first survey of the day occurred between 6 AM and 11:45 AM, the fourth survey between 4 PM and 9 PM, and the EOD survey—administered near bedtime—occurred between 9 PM and 12 AM.

**Figure 1:**
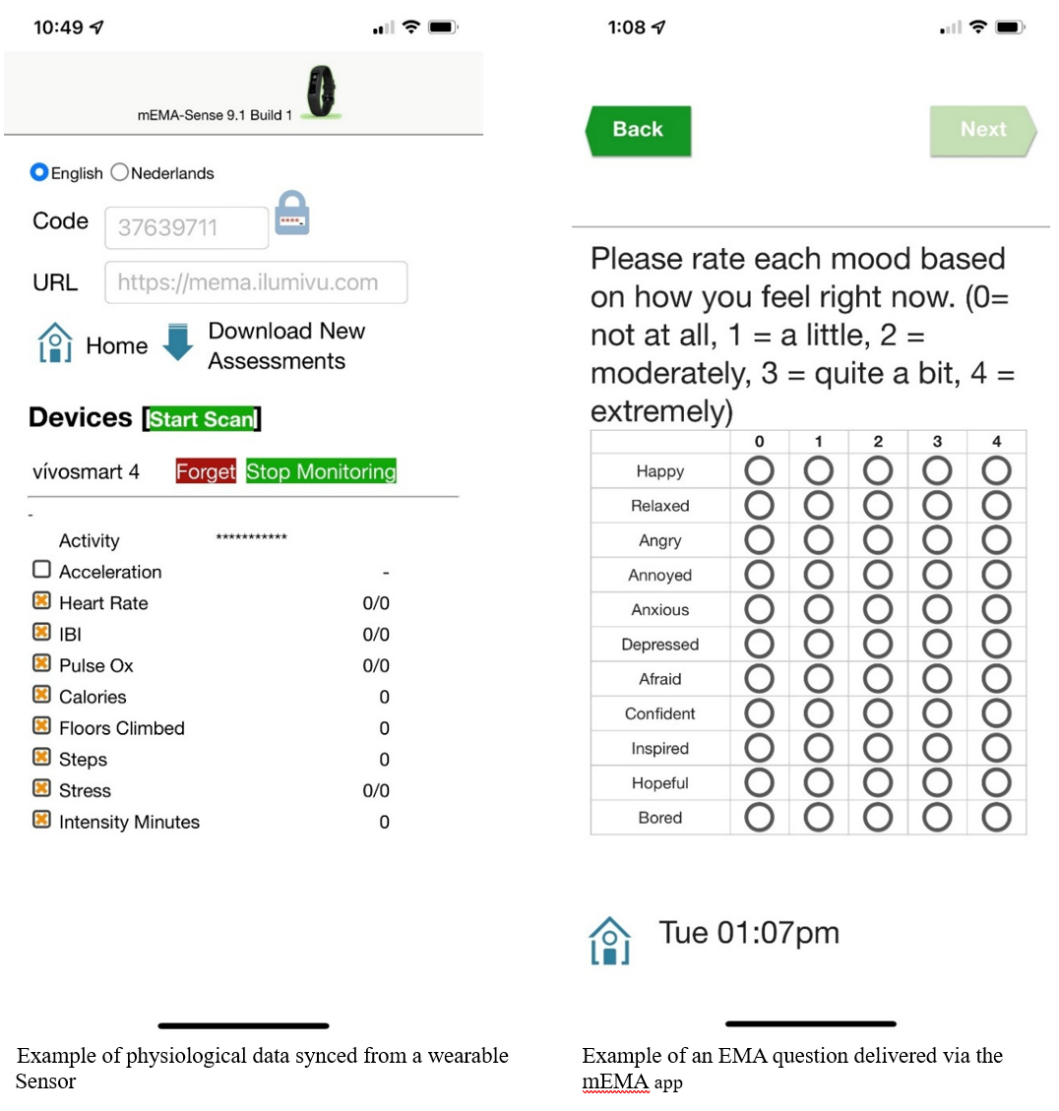
Screenshots of sample ecological momentary assessment (EMA) question and sensor data delivered via the mEMA smartphone app, 2021-2022.

After survey scheduling, a follow-up Zoom meeting was arranged for approximately 2 days after the survey start date to troubleshoot any issues. Participants then began training, which included downloading and syncing the mEMA Sense smartphone application to their personal device. The mEMA app, developed by ilumivu Inc, is available at no cost for iOS and Android platforms. It collects real-time survey data and connects via Bluetooth to the Garmin Vivosmart 4 wearable sensor, which continuously records physiological data such as heart rate and interbeat intervals for HRV estimation. Participants were guided through the process of syncing the wristband with the app and completing test EMA and EOD surveys. Figure 1 displays example screens from the mEMA app interface.

After completing the training session, an in-person exit visit was scheduled for participants to return their device and complete the exit survey. To ensure proper return of the device, participants were presented with a Device Loan Agreement, which they signed, stating that the device was for exclusive use in data collection during the study, was rented on a short-term basis, and must be returned within 7 days of the conclusion of their participation. Additionally, participants were asked if they preferred to receive daily reminders through the Remind app [72], a communication tool for sending messages. All participants agreed to receive a daily reminder text each morning to help them stay on track with their survey schedule. The Remind app was also used to communicate any issues participants encountered during the study period, including scheduling changes or other study-related matters.

The mEMA app was programmed to deliver EMA surveys at 4 participant-chosen times per day during waking hours. The first prompt began within an hour after waking and was followed by 5 push notifications at 5-minute intervals (5, 10, 15, 20, and 25 minutes). If the participant did not respond within 30 minutes, the survey was programmed to be closed and marked as a nonresponse. To minimize participant burden and reduce potential bias, several design features were implemented. EMA surveys were designed to be brief, taking no more than 3 minutes to minimize burden [73], and were limited to 5 times a day. A daily EOD survey—administered before bedtime—assessed stress and substance use experiences not covered in the EMA surveys (Table 1 displays content). After the 14-day data collection period, participants completed a brief exit survey including quantitative and open-ended questions on survey clarity, difficulty, and overall satisfaction with the study protocol. The exit survey took less than 10 minutes to complete (Table S3 in Multimedia Appendix 1 shows details). Responses were analyzed to refine the EMA study protocol for future iterations.

#### EMA Survey Questionnaire and Modification

We established the EMA questionnaire based on a thorough literature review, discussions among our research team, and validation through focus group discussions. Several items we used were originally developed for general survey use; thus, we implemented some adaptations for the EMA context. Detailed questionnaire items and their response options are provided in Table S1 in Multimedia Appendix 1.

Initially, we used the Everyday Discrimination Scale [55] to measure minority stress. However, during the execution of the EMA study, we observed a significant limitation. Relying on the Everyday Discrimination Scale alone to assess minority stress provided an incomplete picture of the multifaceted SGM-related and general stressors encountered by participants. To measure participants’ stress experiences more comprehensively, it was essential to supplement the measurement of minority stress with concurrent assessment of general stress, encompassing daily stressors. Additionally, other measures of minority stress were needed to capture the breadth of the minority stress experiences within this cohort.

Thus, the EMA/sensor study was divided into 2 phases due to the addition of new survey items. The first phase included 6 participants, and the second included 14 participants. Table 1 depicts specific modifications made between these 2 phases and Table S1 in Multimedia Appendix 1 shows details on item wording. Eight additional questions were selected from the sexual orientation microaggression inventory-short form in phase 2 to gauge minority stress related to participants’ SGM status [50]. These questions addressed issues such as anti-gay attitudes and expressions, denial of sexuality, heterosexism, and societal disapproval. We also introduced a time categorization for stressful events, ranging from 0 to 30 minutes ago, 30 to 60 minutes ago, 1 to 2 hours ago, 2 to 3 hours ago, and 3+ hours ago, to enable stressful events to be measured at more proximal time points. Furthermore, 2 general stress measures were also added in phase 2. One measure asked participants to rate their current stress levels on a scale from not at all to extremely [60]. The other measure, the ecological momentary assessment of stressful events by the science of behavior change, encompasses inquiries about stress exposure, stressor type, timing, and intensity [59].

#### Safety Protocol

There is no evidence that repeated measures of stress introduce mental health issues or self-harm behaviors in studies on stress; however, given the high prevalence of mental health concerns among our target population and to minimize any potential harm associated with recurrent stress inquiries, our team implemented a safety protocol aimed at safeguarding the mental well-being of participants in our study.

Upon enrollment, participants received a comprehensive list of mental health resources, which contained information on local and national mental health resources, including the 988 Suicide and Crisis Lifeline. Additionally, a licensed clinical psychologist trained all research personnel who interacted with participants to manage adverse events. This training included instructions on how to implement the safety protocol and respond to different risk levels.

To identify participants who noted suicidal ideation (or other potential harmful situations we would need to respond to right away), we used a feature in the mEMA app that would send an immediate email notification when participants typed in a response to the question noted above. The automated email contained the date, time, and content of the response. Upon receipt of the email notification, the study staff would review the response for signs of intent to harm self or others. In the case that there was cause for concern, an email was sent to the participant asking for a Zoom meeting and informing the principal investigator and a supervising psychiatrist of the situation, along with additional information about the participant and the Zoom meeting.

During the meeting, the team members assessed suicide risk using the Columbia-Suicide Severity Rating Scale [74], gathering details on current thoughts, intent, access to means, protective factors, and past attempts. If risk is identified, the member will briefly consult their supervisor, per study protocol. While keeping the participant on Zoom, the team member mutes their audio/video and contacts the psychiatrist. Based on the psychiatrist’s evaluation, the next steps fall into one of three categories. (1) Emergency: If emergency services are needed, the supervisor joins the Zoom briefly before calling for help while the staff member stays with the participant and engages in calming or distraction activities. (2) High risk (no Emergency): If the participant is high risk but stable, the staff member rejoins the meeting and completes a Safety Plan with them. (3) Low risk: The participant is offered to complete a Safety Plan [75,76], a tool designed for all risk levels to help individuals navigate suicidal feelings and urges, which contains a prioritized list of coping strategies and sources of support that can be used in times of distress. In all cases, the session ends with the participant being provided crisis resources.

We encountered 2 safety incidents during the study. The first incident occurred when a participant completed the EOD survey and responded to the question, “Is there anything else you’d like to report about your day?” and indicated that they had suicidal impulses but did not attempt. As soon as the study team became aware of this event, a team member spoke with the participant via phone to assess their current suicidality and offered the List of Resources for Emotional Distress. The next day, a study team member met with the participant via a Zoom meeting to assess their current mental health status and any suicidal thoughts. The participant stated that they did not have any current suicidality. Following this incident, we voluntarily paused the study and implemented our revised risk assessment protocol described above.

The second incident also involved a participant completing the EOD survey. In response to the same question that triggered the first event, the participant mentioned contemplating suicide without any specific plans or intent. Upon receiving this notification, the study team promptly consulted the clinical psychologist and arranged a Zoom meeting with the participant. The following day, study team members met with the participant via Zoom and assessed the participant’s current risk. Under the clinical psychologist’s guidance, we determined that the participant’s risk was moderate. We then completed the Safety Plan and provided the participant with the mental health resources list.

### Ethical Consideration

All study procedures were reviewed and approved by the IRB at San Diego State University Human Research Protection Program (HS-2020-0016). All participants in the pilot study provided informed consent prior to enrollment, in accordance with IRB-approved protocols. Consent procedures for the EMA/sensor study ensured that participants understood the nature of the study, including the use of wearable sensors and EMA to collect real-time data on stress experiences and physiological responses. For any secondary analyses conducted using data from this study, the original informed consent included provisions for future use of deidentified data, and these analyses were also reviewed and approved by the IRB. No additional consent was required for the secondary use of data.

All data collected during the study were treated with strict confidentiality. Survey responses and physiological data were linked using participant ID codes and stored separately from identifying information. Data were deidentified prior to analysis, and only approved research personnel had access to the key linking IDs to identities. Electronic data were stored on secure, encrypted servers in compliance with institutional data security policies. No personally identifiable information was included in any publications or reports resulting from this research.

Participants could receive up to US $200 in gift cards for full participation in the study, which included attending the initial meeting, completing daily surveys over a 14-day period, returning the wearable device, and completing the exit survey. Compensation was prorated based on the level of participation, with tiered incentives tied to EMA survey completion rates. Participants received a US $40 gift card after the initial meeting, and up to US $160 upon study completion. Those who completed 50%-80% of the surveys received an additional US $30 gift card, and those who completed over 80% received an extra US $30. All participants returned the wearable devices, which were loaned under an agreement that outlined the return policy and potential withholding of incentives if devices were not returned.

### Data Analysis

The feasibility and acceptability of the EMA/sensor study were assessed using participant response rates and responses to the exit survey. For feasibility, adherence was evaluated by tracking participants’ compliance with the study protocol, including the percentage of EMA surveys completed and the number of days participants wore the sensor. We also examined prompt delivery success, overall study retention, and daily trends in response rates (eg, weekday vs weekend completion). Prompt-to-response time was calculated to assess the delay between signal and survey initiation, and survey completion times were compared between the 2 study phases due to the addition of EMA items. For acceptability, participants completed a structured exit survey, in which they rated the relevance, length, and frequency of the EMA surveys using 5-point Likert scales ranging from “completely disagree” to “completely agree.” Additional questions assessed whether the study met their expected level of commitment, whether completing study activities caused added stress, and their willingness to participate in similar studies in the future. We also explored whether EMA response rates differed by demographic characteristics, including age, sex assigned at birth, race/ethnicity, sexual orientation, gender identity, income, and marital status.

## Results

### Demographics

The sample consisted of participants aged 18 to 27 (mean 21.7, SD 2.6) years and included a diverse range of gender identities, sexual orientations, and racial/ethnic backgrounds. Detailed demographic characteristics are presented in Table 3.

**Table 3:** Demographic characteristics of the ecological momentary assessment/sensor study participants of the Stress and Heart Study of LGBTQ+ (lesbian, gay, bisexual, transgender, queer, and other diverse sexual and gender identities) young adults (N=20), 2021-2022.

Category			Total sample (N=20)		Assigned male at birth (N=6)		Assigned female at birth (N=14)
Age (years), mean (SD)			21.7 (2.59)		21.2 (2.23)		21.9 (2.87)
**Race/ethnicity, n (%)**							
	Non-Hispanic White	9 (45)		2 (33.33)		7 (50)	
	Latinx	5 (25)		2 (33.33)		3 (21.43)	
	Non-Hispanic Black	2 (10)		0 (0)		2 (14.29)	
	Non-Hispanic Asian	4 (20)		2 (33.33)		2 (14.29)	
**Sexual** **orientation** **, n (%)**							
	Lesbian/Gay	8 (40)		3 (50)		5 (35.71)	
	Bisexual	2 (10)		1 (16.67)		1 (7.14)	
	Pansexual	4 (20)		2 (33.33)		2 (14.29)	
	Queer	6 (30)		0 (0)		6 (42.86)	
**Gender identity** **, n (%)**							
	Cisgender man	4 (20)		4 (66.67)		0 (0)	
	Cisgender woman	6 (30)		0 (0)		6 (42.86)	
	Transwoman/Transfeminine	1 (5)		1 (16.67)		0 (0)	
	Transman/Transmasculine	2 (10)		0 (0)		2 (14.29)	
	Non-binary	7 (35)		1 (16.67)		6 (42.86)	
**Employment status, n (%)**							
	Employed full-time	5 (25)		2 (33.33)		3 (21.43)	
	Employed part-time	6 (30)		1 (16.67)		5 (35.71)	
	Student	9 (45)		3 (50)		6 (42.86)	
**Marital** **status** **, n (%)**							
	Single	13 (65)		5 (83.33)		8 (57.14)	
	Living with a partner	2 (10)		0 (0)		2 (14.29)	
	Not living with a partner	5 (25)		1 (16.67)		4 (28.57)	
**Income (US $)** **, n (%)**							
	Under 20,000	12 (60)		3 (50)		9 (64.29)	
	20,000-40,000	3 (15)		0 (0)		3 (21.43)	
	40,000-60,000	2 (10)		1 (16.67)		1 (7.14)	
	60,000-80,000	1 (5)		1 (16.67)		0 (0)	
	Prefer not to answer	2 (10)		0 (0)		1 (7.14)	

### Feasibility of EMA/Sensor Study

All 20 enrolled participants completed the EMA/sensor study, including the exit survey, with no attrition. A total of 1400 EMA prompts (5 per day × 14 days × 20 participants) were scheduled and successfully delivered. All prompts were sent as planned, with no delivery failures due to technical issues or participant-related factors. Participants completed 89.4% (1001/1120) of EMA surveys and 92.1% (258/280) of EOD surveys. A total of 18 (90.0%) out of 20 participants completed 80% or more of both EMA and EOD surveys. The average completion rate of all surveys was 89.9% (1259/1400), with a minimum of 70.0% (49/70) and a maximum of 98.6% (69/70). The average response rate across all participants ranged from 83.4% to 93.5% for each day of the week, with lower rates observed on weekends (86.1%) compared with weekdays (91.5%).

We also examined whether response rates varied by demographic characteristics, including age, sex assigned at birth, race/ethnicity, sexual orientation, gender identity, income, and marital status. There was a trend toward higher response rates among racial/ethnic minorities compared with non-Hispanic White individuals (*P*=.057) and among individuals assigned female at birth compared with those assigned male at birth (*P*=.139).

The median time to respond to the prompt signal was 0 minutes, and the average response time was 4.3 minutes. While more than two-thirds of participants responded immediately after receiving the prompt, in some instances, there were delays in response. We had programmed the survey window to close 30 minutes after opening using the mEMA app; however, upon inspection during data analysis, we discovered that the window did not consistently close as intended. In 6.6% (83/1259) of instances, responses were submitted more than 30 minutes after the prompt was issued. This issue appears to be due to a technical limitation of the software. On average, participants took 1 minute and 2 seconds to complete EMA surveys and 58 seconds to complete EOD surveys. Three additional questions were added to the EMA survey after the first 6 participants; as a result, the average completion time was shorter for the first 6 participants (54 seconds) compared with the last 14 participants (65 seconds). Participants wore the sensor for 74.6% (SD 24%) of the expected time (240 hours per person). Seventeen participants (85%) wore the sensor every day, while 3 participants missed at least one day.

### Findings From the Exit Survey

**Figure 2:**
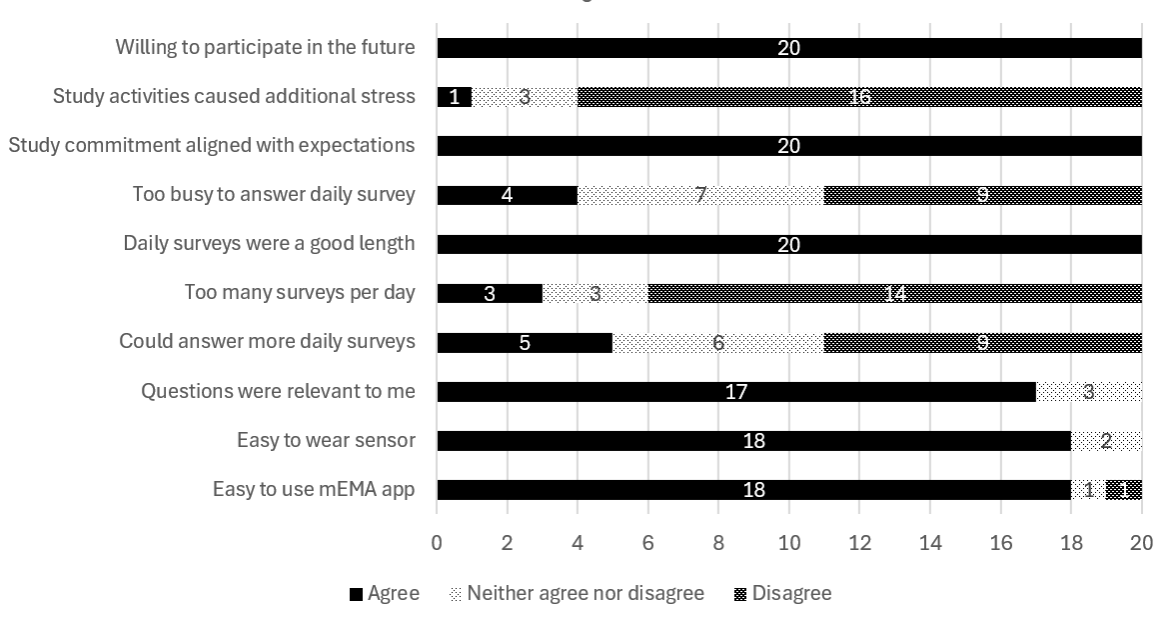
Participants’ exit survey feedback on the feasibility and acceptability of the ecological momentary assessment (EMA)/sensor protocol, stress, and heart study of LGBTQ+ (lesbian, gay, bisexual, transgender, queer, and other diverse sexual and gender identities) young adults (N=20), 2021-2022.

All 20 participants completed exit surveys, with 90% agreeing that the mEMA app and sensor were easy to use. All participants found the EMA questions relevant, felt that the study met their expected commitment level, and expressed willingness to participate in a similar study in the future. Most agreed the survey length was appropriate, the time commitment manageable, and the study did not add stress, though one participant reported additional stress. Full details can be found in Figure 2, and the exit survey questionnaire is in Multimedia Appendix 1.

## Discussion

### Principal Findings

Our study aimed to assess the feasibility and acceptability of using smartphone-based EMA and wearable sensors to evaluate real-time measurements of minority stress experiences and physiological responses among SGM young adults. The high compliance of EMA survey responses and sensor wearing suggests the methodology works well in the target population and can be used for future research. Findings from the exit surveys indicate that participants perceived the study procedures as acceptable and feasible. Most participants reported that the frequency and timing of the EMA prompts and reminders were suitable for the study and their daily lives. In addition, the survey completion time was short, and participants reported that the questions were relevant to their daily experiences and easy to understand. While EMA prompts were programmed to expire 30 minutes after delivery, a technical issue with the mEMA app prevented the enforcement of this time limit. As a result, 6.6% of completed surveys were submitted after 30 minutes. These responses were retained, as they still fell within the broader intended window of assessing experiences since the last prompt. This limitation highlights the importance of verifying software functionality in future EMA studies. The study also encountered some safety concerns, but the study team responded promptly with the established safety protocol. Participants who expressed mental health issues were evaluated and found not to be at high risk for suicidality.

Smartphone-based EMA and wearable sensor studies can face challenges, such as participant burden, noncompliance, and reactivity to the protocol measures. Despite these potential challenges, our study demonstrated an excellent response rate of 89.4% (1001/1120) to the EMA surveys, higher than the average reported in other EMA studies [71,77,78]. In a meta-analysis of 477 EMA studies, these studies, on average, scheduled 6 assessments per day over 7 days, achieving a compliance rate of 79% [71]. Studies with more frequent daily assessments tended to have shorter overall assessment periods. Compliance was not significantly influenced by the number of daily assessments, study design, or sample characteristics but was positively associated with financial incentives. To enhance compliance, we implemented a twofold incentive strategy, which has been used in previous studies [78,79]. In this approach, rewards were distributed after the baseline/training sessions and the exit survey. The reward amount was increased incrementally based on the participant’s response rate. This suggests that this approach to delivering incentives might be an effective strategy for maintaining participant engagement in this target population. Additionally, proactive measures were taken to enhance compliance, including daily morning text message reminders [72] and ongoing communication with participants to address inquiries or concerns. Nonetheless, the small sample size of 20 participants makes it difficult to extrapolate the study’s findings on compliance among all SGM young adults or subgroups.

### Limitations

Wearable sensors also have challenges. Garmin vivosmart4 uses photoplethysmography (PPG), a noninvasive technology that uses a light source and photodetector at the skin’s surface to measure volumetric variations of blood circulation [80-82]. PPG is comfortable and safe for continuous monitoring, which allows for continuous and real-time monitoring of physiological parameters such as heart rate and HRV [83]. However, the accuracy of PPG measurement can be affected by motion artifacts, the fit and position of the device on the body, variations in skin tone, and external factors such as ambient light, temperature, and pressure applied by the sensor [84-86]. The wearable device used in this study was developed for consumer use, not for research, and may not provide accurate heart rate measurements consistently. However, studies to test the validity of the measurement of heart rate with wearables, including previous versions of Garmin devices, have shown acceptable readings [87-89]. Sensor technology has been rapidly improving, and commercial devices are consistently being upgraded. As these advancements continue, the accuracy of these devices will improve, as will the accuracy and reliability of research findings.

Using fixed EMA times selected by participants may have introduced bias if certain time periods were consistently avoided, limiting variability in the data [44,90]. One possible consequence is an underreporting of stress experiences. While random survey timing can lessen this bias by adding unpredictability and better capturing transient events [44], 2 features of our design help mitigate the concern. First, our primary outcome, HRV, was continuously measured by wearable devices and is independent of EMA timing. We calculated the average HRV over several windows (eg, 30 or 60 minutes before each survey and the full interval between surveys); full details of this averaging method will appear in a forthcoming outcome paper. Second, our key EMA variable captured stress experiences since the last prompt, reducing sensitivity to exact survey timing. Future studies may consider randomized prompts within fixed windows (eg, ±30 minutes of a scheduled time) to reduce reactivity while maintaining structure and compliance.

In our study, sensor response rates were lower than EMA response rates. This difference likely stems from the existing use of cell phones versus the addition of a new wearable sensor. EMA surveys were administered through participants’ cell phones, which are indispensable and rarely left behind, resulting in consistently high compliance. In contrast, sensors are a new device to be worn each morning and charged nightly. These additional steps can lead to occasional lapses in use, contributing to greater variability in sensor data collection. We used the ilumivu app for EMA and sensor measurements. For EMA, we monitored the response rate at the end of each day. However, we could only determine if additional data points were collected with the sensor. We could not ascertain the duration of wear until we downloaded and transferred the data into a MATLAB application [91]. During the data management phase, we developed an application to evaluate the sensor response rate daily throughout the study. We plan to use this application in our next study as an aid to improve participants’ frequency of wearing the sensor.

### Conclusion

This study adds to the literature by reporting on the development of an acceptable and feasible research approach that connects real-time minority stress experiences with real-time physiological responses by incorporating EMA methods and sensor technology among SGM young adults. Our goal was to develop a protocol for the study to understand the real-time association between stress experiences and physical health among SGM young adults and assess the feasibility and acceptability of our protocol.

Our findings highlight the feasibility and acceptability of this method. The results demonstrate the suitability of using smartphones and wearable sensors to capture real-time data for assessing the minority stress experiences and physical health of SGM young adults. Furthermore, the findings imply that real-time assessment of minority stress experiences using smartphone apps, along with real-time assessment of stress responses via wearable sensors, has the potential to be an effective tool for future stress interventions. Future research should consider extending the utility of this protocol to more diverse populations, for example, racial/ethnic minorities or economically disadvantaged populations that have health disparities linked to stress exposure.

## Appendix

Multimedia Appendix 1 EMA, End-of-Day, and Exit Surveys Questionnaires.

## Abbreviations

CVD: cardiovascular disease
CVH: cardiovascular health
EMA: ecological momentary assessment
EOD: end-of-day
HIPAA: Health Insurance Portability and Accountability Act
HRV: heart rate variability
IRB: internal review board
LGB: lesbian, gay, and bisexual
LGBTQ+: lesbian, gay, bisexual, transgender, queer, and other diverse sexual and gender identities
NH: non-Hispanic
PPG: photoplethysmography
SGM: sexual and gender minority
TPR: total peripheral resistance

## Data Availability

The datasets generated or analyzed during this study are available from the corresponding author on reasonable request.
