# Supplementary material for: Assessing Minority Stress and Physiological Response Through Ecological Momentary Assessment and Sensors: Protocol for a Feasibility and Acceptability of the Stress and Heart Pilot Study
Source: JMIR Form Res. 2025 Oct 15;9:e68733. doi: 10.2196/68733 (PMC12572751; doi:10.2196/68733)
Supplement: Multimedia Appendix 1 [file formative_v9i1e68733_app1.docx]

| **Multimedia Appendix Table 1. Ecological Momentary Assessment (EMA) Questionnaires** | | |
| --- | --- | --- |
| **Mood** | | |
| Affective emotional states (I-PANAS-SF) | | |
| Leading question |  | Right now I feel... Select all that apply. |
| Items |  | Happy, Relaxed, Angry, Annoyed, Anxious, Depressed, Afraid, Confident, Inspired, Hopeful, Bored |
| Response options |  | Not at all; A little; Moderately; Quite a bit; Extremely |
| **Stressful Events** | | |
| Everyday Discrimination Scale | | |
| Leading question |  | Since your last prompt, have you experienced any of the following discriminatory event? Select all that apply. |
| Items |  | Treated with less courtesy or respect than others  Treated as if others are afraid of you  Treated as if others think you are dishonest or immoral  Treated as inferior, less smart/capable than others  Insulted or called names (direct or overheard)  Received poorer service than others (restaurants, stores, etc.)  Treated unfairly by your family  Stereotyped or negatively labeled  Threatened or harassed  Avoided, excluded, or ignored Have your perspective/feelings overlooked Physically or sexually assaulted  Other |
| Response options |  | Yes; No |
| Follow-up question |  | You selected (present their selection). Is it related to any of the following statuses? Select all that apply. |
| Response options |  | Sexual orientation; Gender identity; Gender; Race/ethnicity; Mental health; Physical health; Appearance; Weight/body size; Religion; Other (Please describe); None/non-applicable |
| Follow-up question |  | When were you treated with (present their selection)? |
| Response options |  | 0-30 min ago; 30-60 min ago;1-2 hours ago; 2-3 hours ago; More than 3 hours ago |
| Follow-up question |  | Where were you treated with (present their selection)? |
| Response options |  | In-person; Online/mobile device |
| Follow-up question |  | You selected In-person. Please describe where it occurred. Select all that apply. |
| Response options |  | Work/school; My home; Other's home; Community space (neighborhood, store, park, gym, bar, restaurant, etc.); LGBTQ community space; Other place (DMV, hospital/clinic, post-office, police station, etc.) |
| Follow-up question |  | You selected online. Please describe where it occurred. Select all that apply. |
| Response options |  | Phone call/text message; Email/direct message; Work/school meeting; Social media (Twitter, Instagram, Reddit, etc.); Dating apps, Online gaming; Online social gathering (Zoom, Google Meet, etc.); Other online space |
| Sexual Orientation Microaggression Inventory (SOMI-SF) | | |
| Leading question |  | Someone said something similar to the following about you or LGBTQ people. (Select all that apply) |
| Items |  | Not to act so gay, butch, queer, etc.  You know how gay people are  Being LGBTQ is just a phase  LGBTQ people don't face discrimination  LGBTQ people overreact when talking about a negative experience related to their sexual orientation or gender identity  I don't mind or have nothing against LGBTQ people, but they shouldn't be so public or open  Being LGBTQ is a sin or immoral  Used the wrong personal pronoun |
| Response options |  | Yes; No |
| Follow-up question |  | When did you hear someone say (present their selection). |
| Response options |  | 0-30 min ago; 30-60 min ago; 1-2 hours ago; 2-3 hours ago; More than 3 hours ago |
| Ecological Momentary Assessment of Stressful Events | | |
| Items |  | Did anything else stressful occur since the last prompt that you haven't mentioned yet? A stressful event is any event, even a minor one, which negatively affected you. |
| Response options |  | Yes; No |
| Follow-up question |  | How would you describe the stressful event(s)? |
| Response options |  | Argument; conflict; disagreement; Financial event; Home-related event; Work-related event; Health event; Event that happened to others; Traffic or transportation event; Other |
| Follow-up question |  | When did the event happen? |
| Response options |  | 0-30 min ago; 30-60 min ago; 1-2 hours ago; 2-3 hours ago; More than 3 hours ago |
| Follow-up question |  | How stressful was the event? |
| Response options |  | Not at all; A little; Moderately; Quite a bit; Extremely |
| General Stress | | |
| Items |  | At the moment, I feel stressed |
| Response options |  | Not at all; A little; Moderately; Quite a bit; Extremely |

| **Substance Use** | |
| --- | --- |
| Alcohol Use | |
| Leading question | Did you drink alcohol? |
| Response options | No; Yes |
| Follow-up question | How many alcoholic drinks did you have? (1 standard drink being one 12 oz. beer/wine cooler, 5oz. glass of wine, one cocktail, or a shot (1.25oz.) of hard liquor.) |
| Response options | 1 or less; 2; 3; 4; 5; 6; 7 or more |
| Follow-up question | What were your reasons/motivations for drinking alcohol? Select all that apply. |
| Response options | Enjoyment/fun/excitement; Coping with sadness/anger/frustration; Coping with tension/stress/anxiety; Relaxation; To socialize/conform socially/peer pressure; Sexual enhancement; It's a habit/compulsive use reasons; It's what was available/easier to get; To feel more comfortable as an LGBTQ individual; To relieve/manage boredom; To manage physical pain; To experiment; Influenced by another substance; Other (Please describe) |
| E-cigarette | |
| Leading question | Did you vape or use an e-cigarette device? |
| Response options | No; Yes |
| Follow-up question | About how many puffs of an e-cigarette did you take? |
| Response options | 1-10; 11-20; 21-30; 31-40 |
| Follow-up question | What were your reasons/motivations for vaping/using an e-cigarette device? Select all that apply. |
| Response options | Enjoyment/fun/excitement; Coping with sadness/anger/frustration; Coping with tension/stress/anxiety; Relaxation; To socialize/conform socially/peer pressure; Sexual enhancement; It's a habit/compulsive use reasons; It's what was available/easier to get; To feel more comfortable as an LGBTQ individual; To relieve/manage boredom; To manage physical pain; To experiment; Influenced by another substance; Other (Please describe) |
| Cigarette Smoking | |
| Leading question | Did you smoke cigarettes? |
| Response options | No; Yes |
| Follow-up question | How many cigarettes did you smoke? |
| Response options | 1 or less; 2; 3; 4; 5; 6; 7; 8; 9; 10 or more |
| Follow-up question | What were your reasons/motivations for smoking cigarettes? Select all that apply. |
| Response options | Enjoyment/fun/excitement; Coping with sadness/anger/frustration; Coping with tension/stress/anxiety; Relaxation; To socialize/conform socially/peer pressure; Sexual enhancement; It's a habit/compulsive use reasons; It's what was available/easier to get; To feel more comfortable as an LGBTQ individual; To relieve/manage boredom; To manage physical pain; To experiment; Influenced by another substance; Other (Please describe) |
| Marijuana | |
| Leading question | Did you smoke or consume marijuana/cannabis? |
| Follow-up question | What were your reasons/motivations for smoking or consuming marijuana/cannabis? Select all that apply. |
| Response options | Enjoyment/fun/excitement; Coping with sadness/anger/frustration; Coping with tension/stress/anxiety; Relaxation; To socialize/conform socially/peer pressure; Sexual enhancement; It's a habit/compulsive use reasons; It's what was available/easier to get; To feel more comfortable as an LGBTQ individual; To relieve/manage boredom; To manage physical pain; To experiment; Influenced by another substance; Other (Please describe) |

**Multimedia Appendix Table 2. End-of-Day Survey**

| Perceived Stress Scale-4 | | |
| --- | --- | --- |
| Leading question | Response options | Follow-up question |
| Today, how often have you felt that you were unable to control the important things in your life? | Never; Almost never; Sometimes; Fairly often; Very often |  |
| Today, how often have you felt confident about your ability to handle your personal problems? | Never; Almost never; Sometimes; Fairly often; Very often |  |
| Today, how often have you felt that things were going your way? | Never; Almost never; Sometimes; Fairly often; Very often |  |
| Are there any other stressful events you experienced today that you would like to report? | Never; Almost never; Sometimes; Fairly often; Very often |  |
| Are there any other stressful events you experienced today that you would like to report? | No; Yes | If you selected yes, please describe the other stressful event(s) you experienced today. |
| Substance Use: Other drugs | | |
| Leading question | Response options | Follow-up question  (Free response) |
| Please select which of the following tobacco products you consumed or smoked today. Check all that apply. | Cigar; Chewing tobacco; Hookah; Other; None | If you selected other for consuming or smoking a tobacco product today, please enter the product(s) below. |
| \| What were your reasons/motivations for consuming or smoking the tobacco product(s)? Select all that apply. \| \| --- \| \| If you selected other for reasons/motivations for consuming the tobacco product(s), please describe those reasons/motivations. \| \| What were your reasons/motivations for consuming chewing tobacco? Select all that apply. \| \| What were your reasons/motivations for smoking hookah? Select all that apply. \| | Enjoyment/fun/excitement; Coping with; sadness/anger/frustration; Coping with tension/stress/anxiety; Relaxation; To socialize/conform socially/peer pressure; Sexual enhancement; It's a habit/compulsive use reasons; It's what was available/easier to get; To feel more comfortable as an LGBTQ individual; To relieve/manage boredom; To manage physical pain; To experiment; Influenced by another substance; Other (Please describe) | \| If you selected other for reasons/motivations for consuming the tobacco product(s), please describe those reasons/motivations. \| \| --- \| \| If you selected other for reasons/motivations for smoking cigars, please describe those reasons/motivations. \| \| If you selected other for reasons/motivations for consuming tobacco, please describe those reasons/motivations. \| \| If you selected other for reasons/motivations for smoking hookah, please describe those reasons/motivations. \| |
| Have you consumed any of the following substances today? Select all that apply. | Prescription medication that are not prescribed to you or in excess of medical instruction;  Club drugs (MDMA/molly, ecstasy, GHB);  Hallucinogens (mushrooms, LSD);  Cocaine;  Opiates (heroin, morphine);  Methamphetamine;  Other;  None |  |
| \| What were your reasons/motivations for consuming prescription medication(s) that are not prescribed to you or in excess of medical instruction? Select all that apply. \| \| --- \| \| What were your reasons/motivations for consuming club drugs (MDMA/molly, ecstasy, GHB)? Select all that apply. \| \| What were your reasons/motivations for consuming hallucinogens (mushrooms/LSD)? Select all that apply. \| \| What were your reasons/motivations for consuming cocaine? Select all that apply. \| \| What were your reasons/motivations for using opiates (heroin/morphine)? Select all that apply. \| \| What were your reasons/motivations for using methamphetamine? Select all that apply. \| \| If you selected other for substances you have consumed, please describe the substance(s) below. \| | Enjoyment/fun/excitement; Coping with sadness/anger/frustration; Coping with tension/stress/anxiety; Relaxation; To socialize/conform socially/peer pressure; Sexual enhancement; It's a habit/compulsive use reasons; It's what was available/easier to get; To feel more comfortable as an LGBTQ individual; To relieve/manage boredom; To manage physical pain; To experiment; Influenced by another substance; Other (Please describe) | \| If you selected other for reasons/motivations for consuming prescription medication(s) that are not prescribed to you or in excess of medical instruction, please describe those reasons/motivations. \| \| --- \| \| If you selected other for reasons/motivations for consuming club drugs (MDMA/molly, ecstasy, GHB), please describe those reasons/motivations. \| \| If you selected other for reasons/motivations for consuming hallucinogens (mushrooms/LSD), please describe those reasons/motivations. \| \| If you selected other for reasons/motivations for consuming cocaine, please describe those reasons/motivations. \| \| If you selected other for reasons/motivations for using opiates (heroin/morphine), please describe those reasons/motivations. \| \| If you selected other for reasons/motivations for using methamphetamine, please describe those reasons/motivations. \| \| If you selected other for reasons/motivations for consuming this substance, please describe those reasons/motivations. \| |

**Multimedia Appendix Table 3. Exit Survey Qustionnaire**

**Exit Survey—Stress and Heart Study**

Thank you for participating in the Stress and Heart Study. We value your participation and feedback as we continue to develop this tool for future research. As you finish your participation in this study, we have some questions we hope you will answer. We appreciate your open and honest feedback. There are no right or wrong answers.

**Please indicate how much you agree with the following statements about the daily surveys.**

|  | Completely disagree (1) | Disagree (2) | Neither agree nor disagree (3) | Agree (4) | Completely agree (5) |
| --- | --- | --- | --- | --- | --- |
| The questions in the daily surveys were relevant to me |  |  |  |  |  |
| I could answer more daily surveys throughout the day than were given |  |  |  |  |  |
| There were too many surveys per day |  |  |  |  |  |
| The daily surveys were a good length |  |  |  |  |  |
| I was too busy to answer daily surveys frequently |  |  |  |  |  |

**Please indicate how much you agree with the following statements about the smartwatch sensor.**

|  | Completely disagree (1) | Disagree (2) | Neither agree nor disagree (3) | Agree (4) | Completely agree (5) |
| --- | --- | --- | --- | --- | --- |
| It was easy for me to wear the sensor |  |  |  |  |  |
| It was easy for me to use the mEMA app |  |  |  |  |  |

**Please indicate how much you agree with the following statements about the Stress and Heart Study.**

| Overall, the study was at the commitment level that I expected |  |  |  |  |  |
| --- | --- | --- | --- | --- | --- |
| Completing all the study activities caused additional stress |  |  |  |  |  |
| I would be willing to participate in a similar survey in the future |  |  |  |  |  |

Thank you for answering these questions. We would love the opportunity to chat with you about your experience so that we can improve on our methods in the future.

1. How was your experience for the last two weeks? How did you feel in your day-to-day life while participating in the study compared to the days when you didn’t participate in the study?
2. What [other] challenges or barriers did you experience using the survey mEMA app?
3. What [other] challenges or barriers did you experience using the smartwatch sensor?
4. What did you like about participating in the study? [Is there anything else you would like to share about your experience participating in the study that was positive?]
5. Would you recommend participating in the study to your friends? If you were to recommend participating in the study to your friends, is there anything you would tell them beforehand about the study and your own experience?
6. Are there any specific survey questions that you would like to change? If so, what were they and how would you like to change them.
7. Are there any specific survey questions that did not fit your situation? If so, how do you like to change them?
8. Is there anything else you want to share about your experience participating in the Stress and Heart Study? We welcome any and all feedback.
